# Deep learning for discovering pathological continuum of crypts and evaluating therapeutic effects: An implication for in vivo preclinical study

**DOI:** 10.1371/journal.pone.0252429

**Published:** 2021-06-14

**Authors:** Dechao Shan, Jie Zheng, Alexander Klimowicz, Mark Panzenbeck, Zheng Liu, Di Feng

**Affiliations:** 1 Global Computational Biology and Digital Sciences, Boehringer Ingelheim Pharmaceuticals, Ridgefield, Connecticut, United States of America; 2 Immunology and Respiratory Disease Research, Boehringer Ingelheim Pharmaceuticals, Ridgefield, Connecticut, United States of America; Taipei Medical University, TAIWAN

## Abstract

Applying deep learning to the field of preclinical in vivo studies is a new and exciting prospect with the potential to unlock decades worth of underutilized data. As a proof of concept, we performed a feasibility study on a colitis model treated with Sulfasalazine, a drug used in therapeutic care of inflammatory bowel disease. We aimed to evaluate the colonic mucosa improvement associated with the recovery response of the crypts, a complex histologic structure reflecting tissue homeostasis and repair in response to inflammation. Our approach requires robust image segmentation of objects of interest from whole slide images, a composite low dimensional representation of the typical or novel morphological variants of the segmented objects, and exploration of image features of significance towards biology and treatment efficacy. Both interpretable features (eg. counts, area, distance and angle) as well as statistical texture features calculated using Gray Level Co-Occurance Matrices (GLCMs), are shown to have significance in analysis. Ultimately, this analytic framework of supervised image segmentation, unsupervised learning, and feature analysis can be generally applied to preclinical data. We hope our report will inspire more efforts to utilize deep learning in preclinical in vivo studies and ultimately make the field more innovative and efficient.

## Introduction

Histology data derived from tissues is indispensable for evaluating drug efficacy in preclinical intervention studies. We used Dextran Sodium Sulfate (DSS) induced colitis models in our feasibility study to evaluate epithelial-based histological healing. Crypts of Lieberkuhn are moat-like invaginations of the epithelium around the villi. The stem cells from the invaginations’ base continually divide and provide the source of epithelial cells in the crypts and villi. Cryptitis and crypt distortion are often described as pathological features of human inflammatory bowel disease (IBD). Ideally, a successful drug intervention will prevent crypt loss and restore epithelial cell dynamics.

In the past, endeavors have been made to semi-quantitatively assess epithelial tissue injury or healing, which have been used for preclinical model-based efficacy investigations [1–3]. For colitis models, some researchers have used histological injury scores based on the proportional damage within crypts [4, 5]. Other methods [6, 7] are based on pathological changes including hyperplasia and goblet cell reduction [8]. Current proposed histology scoring systems include crypt features as one of the components. A recent report of crypt associated injury and regeneration suggests the morphological variation in crypts are associated with the homeostasis-injury-regeneration dynamic [9]. Despite efforts to develop improved scoring systems to assess the crypts, the issue is that current scoring systems use labor intensive manual scoring that is coarse-grained, and discord among experts is common [10]. Currently, there is a lack of both reports and common consensus on the morphological changes of crypts observed under therapeutic intervention. To address these issues, we present a digital pathology workflow to detect and segment crypts from WSIs, followed by extraction of minable features used for pre-clinical efficacy screening.

Image segmentation of crypts contains challenges due to the morphological variation related to the injury-regeneration dynamic. Both classical computer vision processing and deep learning based methods have been successfully used for image segmentation in the past. Segmentation of glandular structure can be achieved by color-gradient-based, morphology-based [11, 12], or graph-based methods. Multiple convolutional neural network (CNN) architecture [13–15] based models have been proposed for Gland Segmentation in the Colon Histology Images, thanks to the GlaS challenge [16]. Most of the deep learning based segmentation models share the core idea of using a down-sampling path and an up-sampling path. CUMedVision, the top model selected from the GlaS challenge, is a multi-level contextual fully convolutional network (FCN) that requires post-processing of probability maps, including smoothing with a disk filter and the radius, filling holes and removing small spurious segments [17]. Feature Pyramid Network (FPN), which relies on pyramid feature maps over the input image for feature extraction at multiple levels, has been reported to segment pixels at borders within clustered objects such as cell clusters [18]. The advantage of this architecture is to take dual signals such as cell and nucleus for fluorescence microscopy images [19]. It also requires post-processing steps to improve the results. Pyramid scene parsing network (PSPNet) takes into account the context of the whole image to predict the segmentation [20]. Finally, U-Net, a competitive model, has been widely used for biomedical segmentation without complicated post-processing steps.

The next challenge for pre-clinical study is that unseen variations of cells or cellular organization may occurs in the new experimental disease models. A new score system will be unlikely generated. Direct examination of all ten thousand crpyts from high resolution WSIs is impossible for humans, which is why a new strategy is needed. Ideally, we define and characterize the pathological state based on histologic structural similarities. This goal can be achieved by the construction of latent space representations of our segmented images, as latent space visualization techniques allow humans to observe a massive number of the segmented objects in one two dimensional (2D) map. To construct our visualization, we decided to use an autoencoder (AE), which performs a non-linear dimensionality reduction with the ability to learn non-linear manifolds. We demonstrated that unsupervised learning can create representations of sets of segmented crypts with different pathological states.

**Fig 1:**
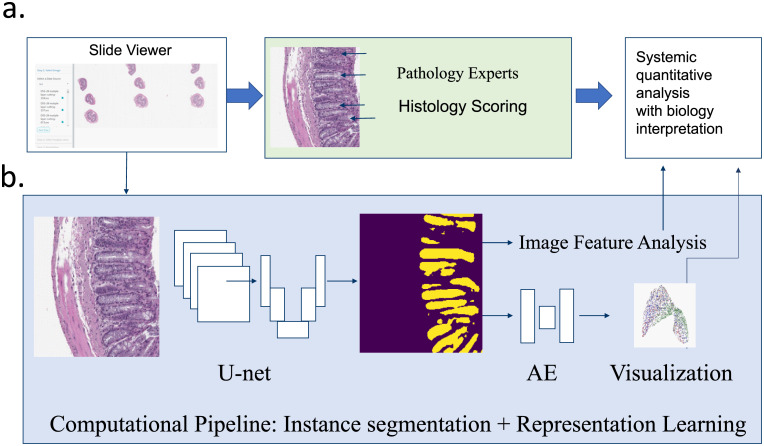
Comprehensive assessment of crypt microstructural changes during tissue injury and recovery **a**. standard of protocols to evaluate WSIs using software assistant semi-quantitative assessment. **b**. Deep learning augmented computational workflow to process digitalized WSIs across whole tissue fields with various localization.

Current digital pathology tools allow us to measure the number and area of crypts, which are then interpretable by human practitioners. On the other hand, use of more detailed non-linear information, which can be encoded by statistical features, is relatively rare, due to its difficulty for practitioners to interpret. However, non-linear information can still be extremely useful. In this study, we present an end to end workflow (Fig 1) to show that texture-based statistical features can help to quantitatively evaluate the effects of drug treatments on the epithelial tissue recovery. Our feasibility study suggests that a deep learning based approach can accelerate the evaluation of preclinical studies from weeks to hours.

## Materials and methods

### Datasets description

Archived images with Hematoxylin and eosin stain (H&E) were retrieved for reanalysis from one study pertaining to colitis models. Description of DSS induction protocol: Female C57BL/6 mice weighing 21-24g were obtained from the Charles River and allowed to acclimate for a minimum of one week prior to experimentation. Mice were kept on a 12 hour light dark cycle and provided access to food and water ad libitum. DSS of 36,000–50,000 polymer MW (MP Biomedical, 160110) was dissolved in water at a concentration of 3% w/v. On study day 0, mice were randomly assigned to groups and given 3% DSS solution in water bottles. DSS supplemented drinking water was provided for a total of five consecutive days. Mice were treated with sulfasalazine (Spectrum Chemical Mfg.) at 600 mg/kg or solvent by oral gavage. All treatments were administered twice daily for the duration of the experiment. A separate group of untreated naïve mice were provided with water only. Mice were humanely euthanized on day 7 to collect tissue samples. Distal and middle sections of colon were collected, fixed in 10% formalin for 24 hours, transferred to 70% ethanol, and processed with standard preclinical protocols for tissue processing, embedding, cutting, and H&E staining. All experiments were conducted in compliance with the rules set forth by the Boehringer Ingelheim site Institutional Animal Use and Care Committee (IACUC) in accordance with the guidelines established in the National Institutes of Health, Guide for the Care and Use of Laboratory Animals. The study was conducted in Boehringer Ingelheim’s AAALAC (International, Association for the Assessment and Accreditation of Laboratory Animal Care) accredited facility. The experimental protocol was reviewed and approved by the site’s IACUC. Experimentation was conducted with consideration to minimize subjects’ pain and distress. Euthanasia was performed in accordance with the guidelines established in the Panel on Euthanasia of the American Veterinary Medical Association.

### Digital image acquisition

Multiple WSIs were scanned 20X (0.5μm/pixel) using Leica Aperio AT 2 scanner. After detecting the part of the image that contains the matter from WSI, all pixels foreground from each WSI were extracted and saved as images. The tissue size from each slide ranges from 3000–6000 pixel. The naïve group contained 73 total WSIs from 12 individual animals; each sample had 4–7 WSIs. 17271 glands were segmented from this group. The DSS group contained 61 WSIs from 11 individual animals. 8070 glands were segmented from this group. Gland loss is a hallmark for the DSS induced colitis. For the treatment group, we had 76 WSIs derived from 12 samples. Total number of segmented glands were 14317.

### Training and validation set generation

To improve the training efficiency, we relied on random image tiling, followed by image augmentation strategies to expand the training image sets to improve the model performance. Training image tiles were generated using a sliding window approach applied on annotated WSI. The width and height of the window size were random integer numbers between 256 to 768 pixels. The stride length ranged from 256 to 512 pixel. For each round of training tile generation, a new set of 100 tiles were generated from each annotated WSIs. These tiles were resized to 512x512 pixel, which is the input size for the network.

### Image augmentation

We generated augmented images before training the segmentation model. Typically, a combination of affine transformations was used to randomly crop and resize image, apply gaussian blur with random kernel, and rotate with random angle. Random resized cropping or spatial augmentation is used to make model robust to changes in the scale and position of segmentation objects [21]. The crops of random size of the original size in range 256–768 with a random aspect ratio of the original, followed by final resize to 512x512 (S1A Fig in S1 Appendix). We use this cropped window to scan the WSI with a random stride using integer number between 256 to 512. Therefore, total number of tiles generated after scanning from each slide reach about 100. For a training tiles dataset of size N, we can create a synthetic dataset of 5N. For each round of training, we used total 800 tiles generated from all 8 annotated WSIs. After five rounds, a total 4000 tiles can be used. Color augmentation is optional and may be used if datasets are affected by multiple batches, fixation condition, overstaining or understanding. As shown in S1B Fig in S1 Appendix, we found that spatial augmentation improves the validation accuracy using Glas dataset. We included the results of accuracy curve and metrics using Glas dataset in the supplement document. We only applied this during training.

### Crypts segmentation

**Fig 2:**
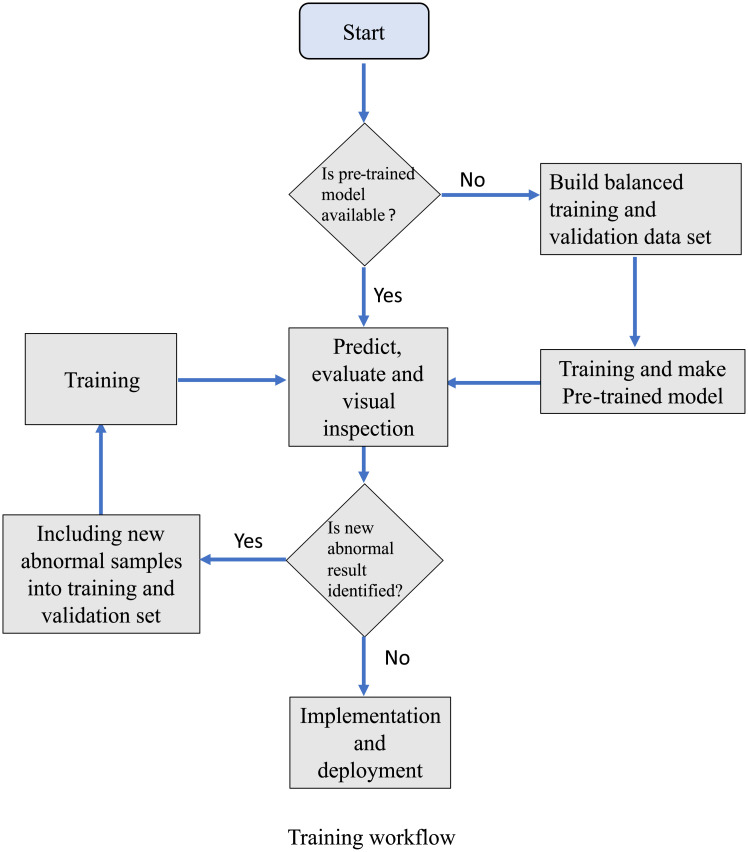
A training workflow for histopathology lab. The decision make step is shown in text with question marker. The arrows indicate the direction. After making the basic pre-trained model, the training can be implemented using quick annotator or equivalent.

**Fig 3:**
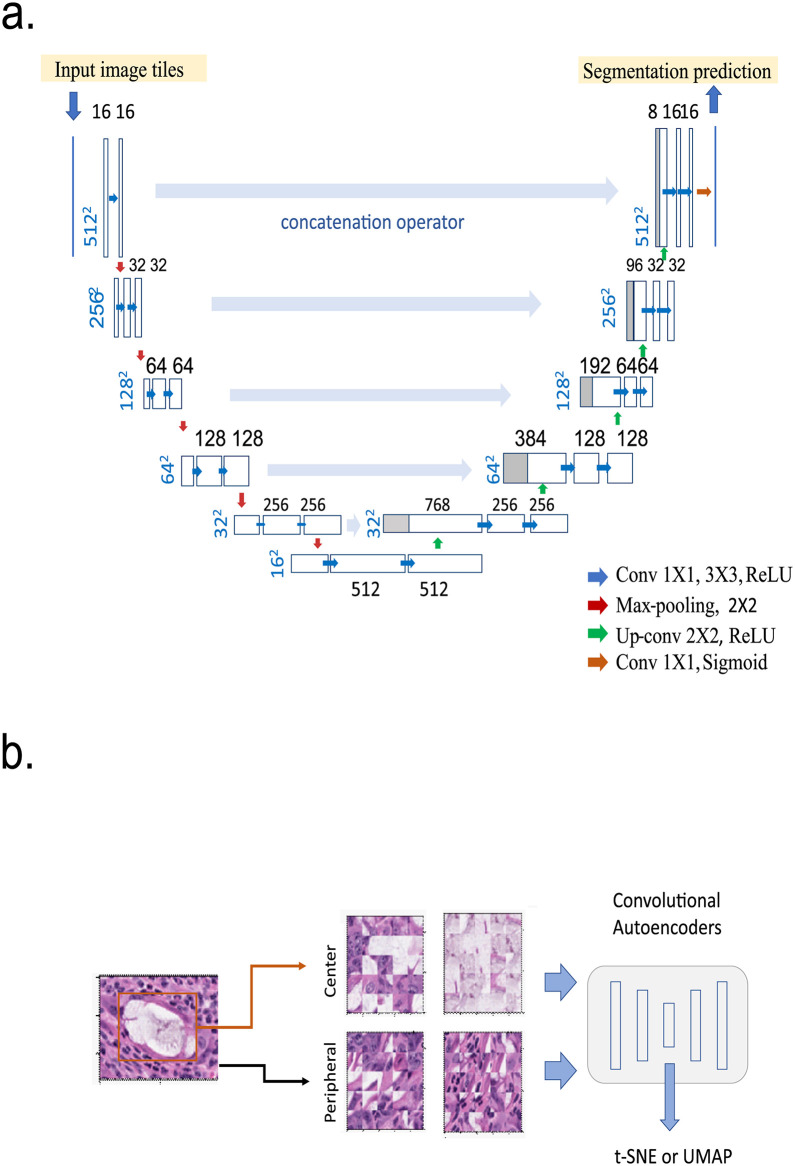
a. Architecture of Multichannel Convolutional Networks used for Crypt Segmentation. The boxes represent cross-sections of square feature maps. Number of map size dimensions were annotated on the lower left, and the number of channels were labeled on the top of the correponding box. The leftmost input were 512×512 image tiles sampled from the whole slide. The rightmost of the output is the CNN’s binary ring mask prediction. Arrows represent operations and convolutions, b. Architecture of Autoencoder Network includes 4 steps. Step 1: the leftmost map represents gland patches generated from Convolutional U-Net. Step 2: Random extraction of the 24x24 patch from central and peripheral regions of the gland patch. Step 3: multiple channel convolutional autoencoder architecture. Step 4: t-SNE visualization using data from bottom neck.

We used U-Net as our network architecture (Fig 2a). The input image tiles and their corresponding segmentation maps from the annotated collection are used to train the network. All images, including augmented images, were used to feed the network during the training, but only the untransformed original images were used for validation sets. We split 80% for training, 20% for validation. After we created this pre-trained model, we continue to improve the model by bootstrapping annotations using Quick Annotator’s approach [22] to accept to correct erroneously segmented structures identified from new samples. The overall training workflow is illustrated in Fig 3. For accurate prediction, the overlap-tile strategy was used to process the output result (512×512 pixels). For each output tile, we only used an area (480x480 pixels) from the center of the predicted results. To predict the pixels in the border region of the image, the missing area was extrapolated. We performed seven rounds of training. F1 and Dice score were used to evaluate the segmentation model.

### Representative learning of crypts using convolutional autoencoder

In this task, our goal is to distinguish or identify morphological variants of the crypts. Specifically, we are interested in variation in internal structures such as cellular component and secretory content within each crypt. The variation in the shape and sizes of the crypts itself is not important in this task. Therefore, we need to focus on the central and peripheral region that contain important internal structural of the crypts. As shown in Fig 2b, we sample both the central region and the peripheral border of each segmented crypt to feed the multiple channel autoencoder. For each of the segmented crypt image, 24 patches (24×24 pixels) were randomly sampled from the central region and peripheral border of the segmented image. The input size is 24×24 pixels with a total of 72 channels (3 RGB×24), for the autoencoders. The detailed parameters were shown in S1J Fig in S1 Appendix. In the encoder network, the model downsamples 4 times. In the decoder network, the model upsamples 4 times back to 24 x 24 x 36. We obtained the encoded feature space from bottleneck, followed by t-SNE to generate a 2D scatterplot. We used ReLU as the activation function, binary crossentropy as the loss function and adam as the optimizer. For padding options, output have the same length as input. Filter sizes of the combined autoencoders are 96, 64, 32, 24 in the encoder layers and 24, 32, 64, 96 in the decoder layers.

### Image feature

To include the spatial interaction between image pixels for analysis, we used a Gray Level Concurrence Matrix (GLCM) model to extract second-order statistical features for the derivation of the crypts’ textural measure. Based on the segmented crypts and mucosa image patches, we compute the GLCM texture features including Energy, Contrast, Entropy, Homogeneity, Correlation, Dissimilarity, and Angular Second Moment (ASM), as well as Entropy, Canny, and Energy.

### Histology score

The epithelial scores and inflammation score were generated using a modified method based on traditional histology scoring [17]. HALO software was used to help examing the slide and quantifying epithelial scores and inflammatory scores by two pathologists. We used quick annotator equivalent tools to annotate images. Image annotation was done by drawing and lines editing.

### Statistical and computational methods

The HistoQC, the OpenCV python library and skimage were used to check quality, analysis, and compute the image features and morphometric value from the segmented images. R and Python were used for analysis and plot. Python library with Neural Networks for Image Segmentation used for model comparison was from https://github.com/qubvel/segmentation_models.

## Results

### Segmentation

**Fig 4:**
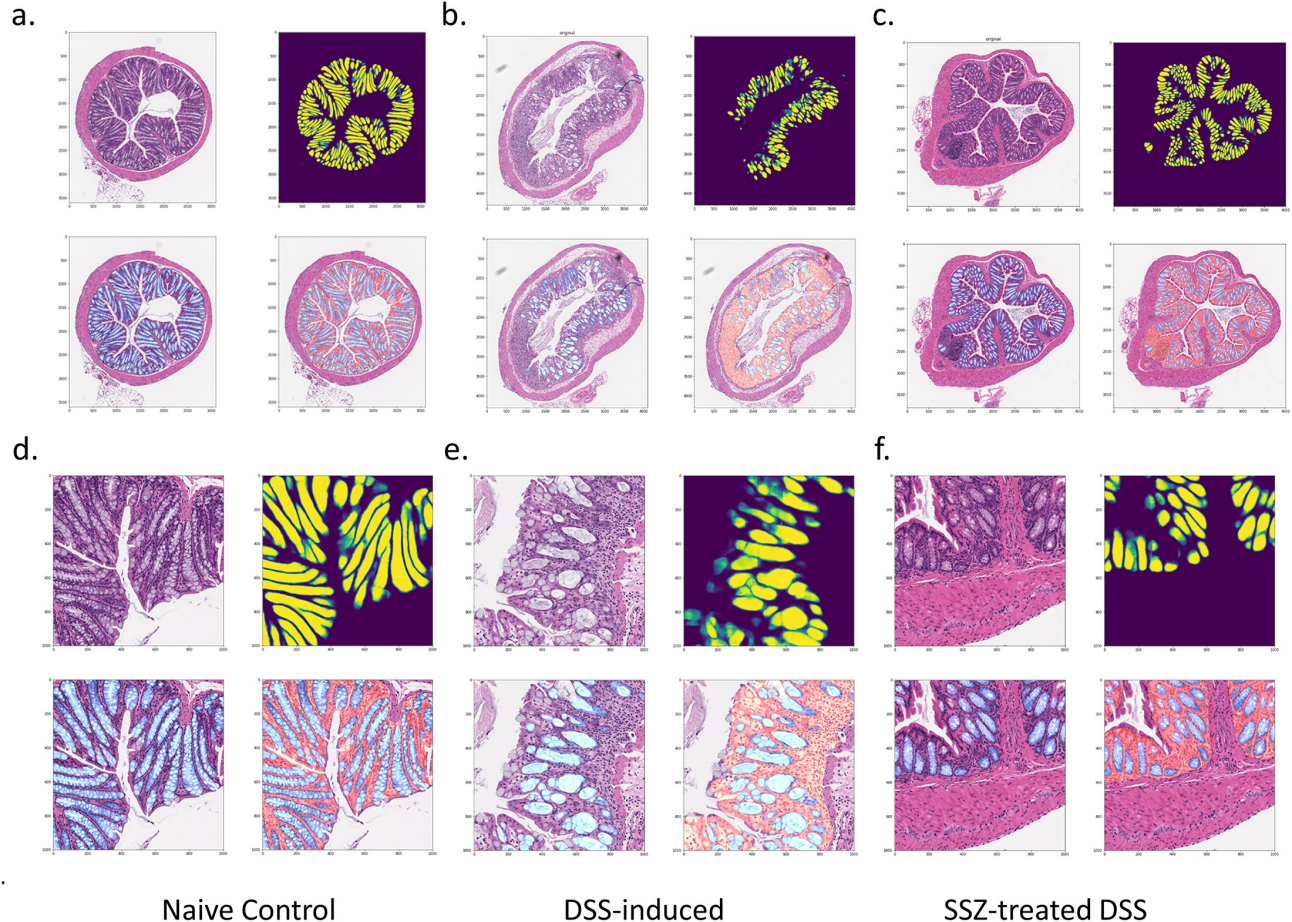
Segmentation results from the mouse colon tissue dataset. Whole image of a. Control colon b. colon from DSS model, c. colon from treated DSS model. Details of b. Control colon c. colon from DSS model, d. colon from Sulfasalazine SSZ treated DSS model. For each panel, upper left: Original H&E image. Upper right: Predicted probability mask of gland (pixel level). Lower left: H&E overlaid with crypts binary mask. Lower right: H&E overlaid with predicted masks of gland (blue) and mucosa (orange red).

Applicable machine model for in vivo study requires a balance between prediction speed and precision. We used U-Net, an architecture used for semantic segmentation, to locate crypts from WIS. A more sophisticated model such as mask R-CNN may perform the task well, but in exchange requires a longer time in training and prediction. Our implementation of the U-Net model in this paper achieved much more than 90% validation accuracy and runs fast. Typically, it will take 5 minutes to complete tile generation, prediction, and tile overlapping in AWS G3 instance. We compared the result from U-Net to the reported specialized gland segmentation model from GlaS Challenge Dataset Evaluation using the same datasets and found that U-Net achieved the comparable or better F1 score and DICE score reported by the top models (S1C Fig in S1 Appendix). We also compared U-Net with other most widely used model architectures including FCN-8, FPN, and PSPNet. As shown in S1D Fig in S1 Appendix, all models had resnet18 as backbones and sigmoid as activation function for binary classification. As is clear from S1E–S1H Fig in S1 Appendix, the accuracy and F1score curve indicated no other model being test is superior to U-Net in validation test. Therefore, we adopted U-Net as our model used for segmentation. Fig 4 illustrates the segmentation results from the naïve, DSS, and SSZ treatment group. In comparison to normal colonic crypts from the naïve group, one can clearly see that the DSS-induced group massively lost crypts, as shown in Fig 4b and 4e. After treating these groups with SSZ, these groups usually contained more crypts than non-treated DSS groups.

### Representative learning of crypts to discover morphology pattern

**Fig 5:**
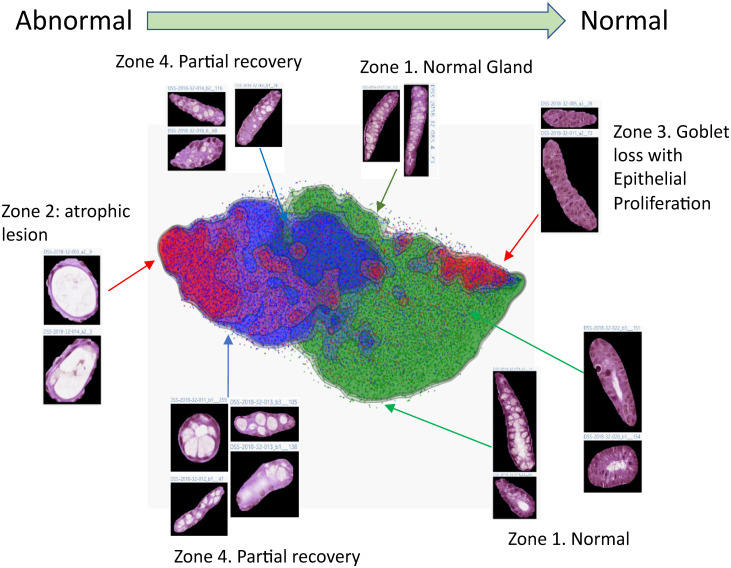
Visualization of a dense representation of all the crypts in the study. The convolutional autoencoder is built to learn representative of segmented glands patches obtained from U-Net. A t-SNE was shown with the typical gland image patches to exemplify the difference. Each data point represents one complete crypt image patch. Total 39655 segmented image of crypts were used in the map.

One of the challenges of disease models is that histological manifestation differs in different animal models. Neither comprehensive pathology information nor pathological structure annotation exist. This makes supervision-based classification impractical. Therefore, we proposed the use of unsupervised learning methods to represent individual crypts in a two-dimensional map. For implementation, we used a convolutional autoencoder to construct a dense representation of the images of segmented crypts. As shown in Fig 5, a 2D T-distributed Stochastic Neighbor Embedding(t-SNE) map can help with visualizing the latent space representation, which reflects the continuum of morphological variants across the Naïve, DSS, and treatment groups. We observed several visually discernable patterns. Zone 1 contains crypts with normal glandular structure, and the columnar epithelial cells were arranged in order. Zone 2 contains atrophic or degenerating crypts, and these crypts had a reduced number of goblet and epithelial cells, which exhibited squamous morphology. Zone 3 contains crypts with predominately epithelial cells without mucin secretion, but no colonic goblet cell differentiation. Finally, Zone 4 contains crypts in partial recovery due to the SSZ treatment. It is noted that the atrophic gland in Zone 2 was reported only very recently, relative to the long history of DSS model in studies and applications. This exciting development reveals the potential that the unsupervised approach has. That is to say, it may allow us to discover new, hitherto unknown histological changes in in-vivo models, which prior knowledge and classification methods have missed.

After we project the group information into the map, the majority of crypts from normal colon tissue are clustered in the right(Zone 1, green), most crypts from the DSS group are clustered in the left(Zone 2, red), and crypts from the SSZ treatment group make up the middle of the map and are partially co-localized with the DSS group(Zone 3, blue). Notably, within the SSZ treatment group, the crypts with less cells were located closer to the DSS group, and the crypts with more epithelial cells were located further away. Both the DSS and treatment groups contained crypts with a marked loss of goblet cells. However, in comparison to the DSS group, the crypts from the treatment group had more epithelial cells. Additionally, it was observed that within the treatment group, while both epithelial and goblet cells had recovery, the recovery rate for epithelial cells was higher than that of goblet cells at the time that the sample was collected. Taken together, the overall pattern in data manifold indicates that the crypts’ morphology after treatment deviated from the disease group toward normal, undiseased crypts. This pattern indicates that the SSZ treatment is effective.

### Image features correlation

**Fig 6:**
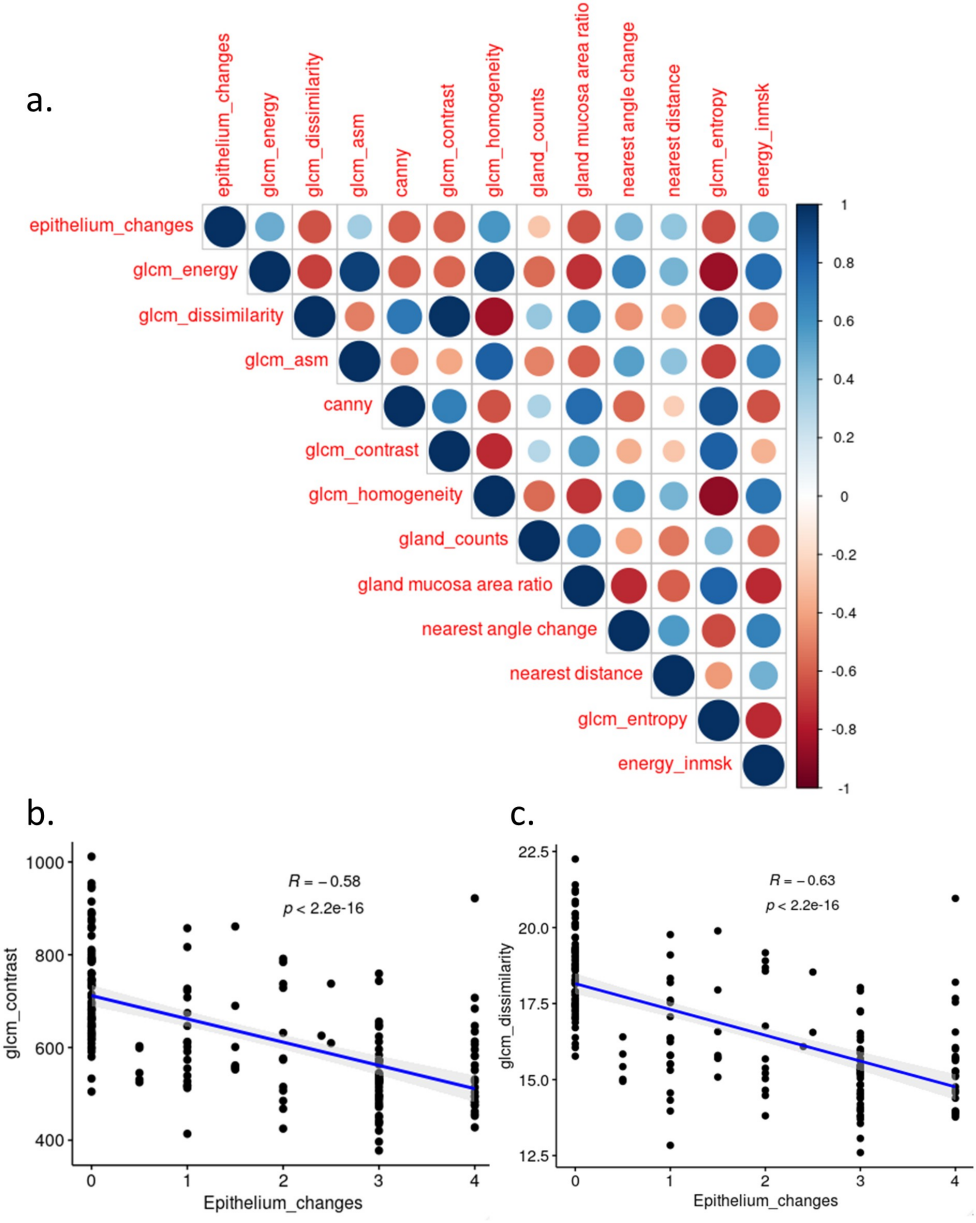
Summary of image features for histology assessment at slide level. A total number of 186 slides were used. a. Correlation map for texture features including histopathologic derived epithelium changes score by pathologist, entropy in masked gland, distances between the nearest glands, and the angle difference between the two nearest glands, GLCM (ASM, homogeneity, entropy, energy, dissimilarity, correlation, glcm_contrast). Agreement between histopathologic derived epithelium change scores and segmentation-derived entropy measures. b. Correlation scatter plot of GLCM contrast with epithelim change score. Each dot represents one individual slide. c. Correlation scatter plot of GLCM dissimilarity with epithelial change score. Each dot represents one individual slide. Pearson correlation coefficient and p-value were shown.

We are interested in identifying features correlated or anti-correlated with the most epithelial score previously used by histopathologists in practice. After correlating image features with epithelial score (Fig 6a), we found the texture features such as GLCM contrast and dissimilarity (Fig 6b) values appear to negatively correlate with the epithelial score provided by pathologists within the SSZ treatment group. While GLCM Homogeneity and GLCM ASM has the positive correlation with epithelial score.

### Evaluation of epithelial healing using image features

**Fig 7:**
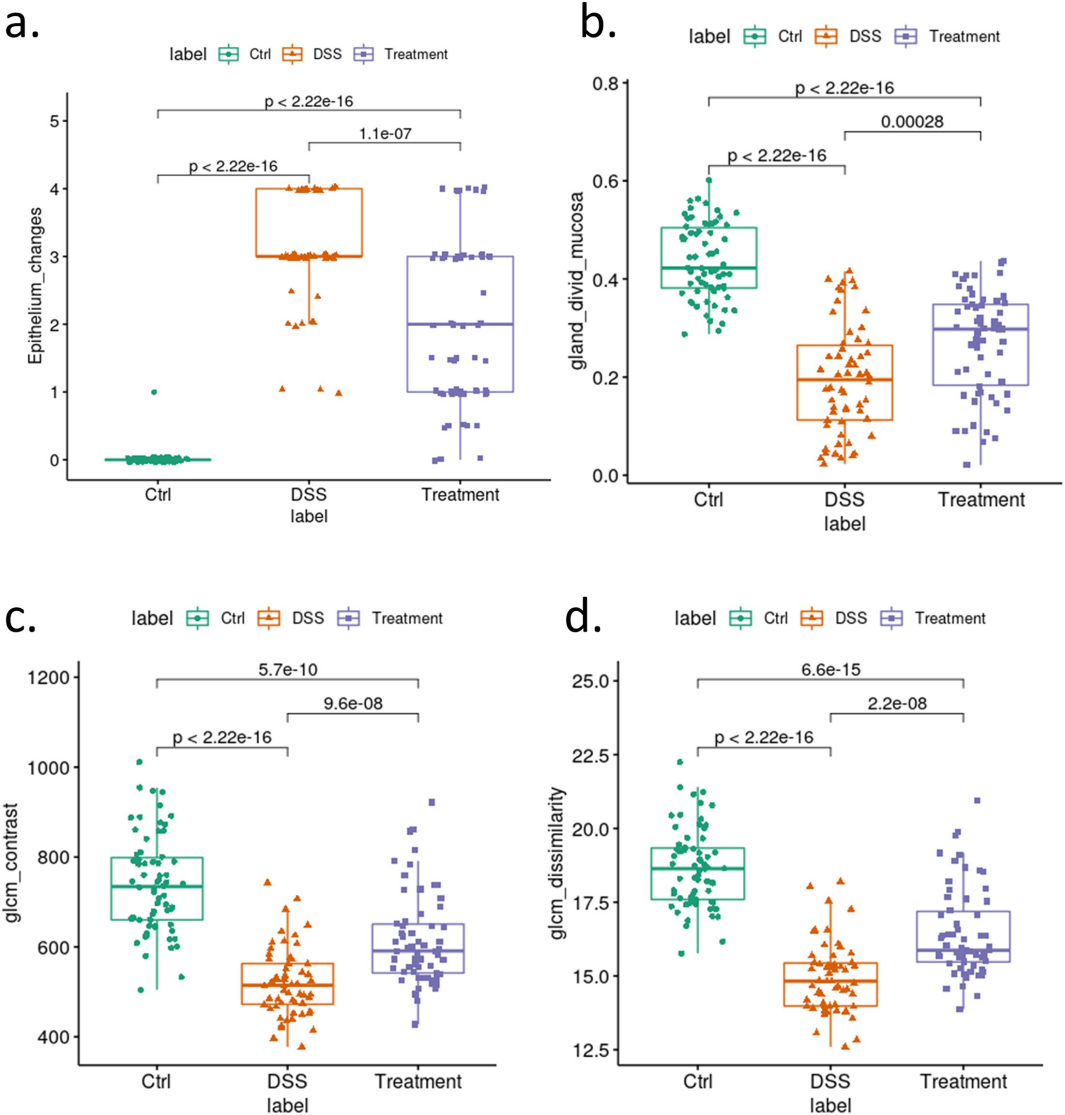
Group comparison among Ctrl (Naïve animal), DSS (DSS-induced colitis), and Treatment (colitis treated with SSZ) at individual slide level using a. histopathologic derived epithelium change score; b. gland and mucosa ratio score; c. GLCM contrast value in masked crypts; d. GLCM dissimilarity value in masked crypts. Data were summarized for individual slides. Each data point represents the average value extracted from all segmented crypts from each slide. All statistical analyses between groups were performed using unpaired two-sided Student’s t tests with unequal variance.

The epithelium change (Fig 7a) were scored by histopathologists to show the epithelial healing in DSS group. From the computation, it was determined that the DSS group had a reduced number of crypts compared to the control, and the crypts from the DSS group had a lower gland/mucosa area ratio (Fig 7b). Analysis of the above features within the treatment group indicates that the treatment causes partial recovery. As expected, the epithelium change score and gland/mucosa ratio decreased with a significant p-value in the DSS group and increased with a significant p-value in the DSS+SSZ group (Fig 7b). The loss of normal crypts’ histological architecture in the DSS group decreased the value of GLCM contrast (Fig 7c) and dissimilarity (Fig 7d).

**Fig 8:**
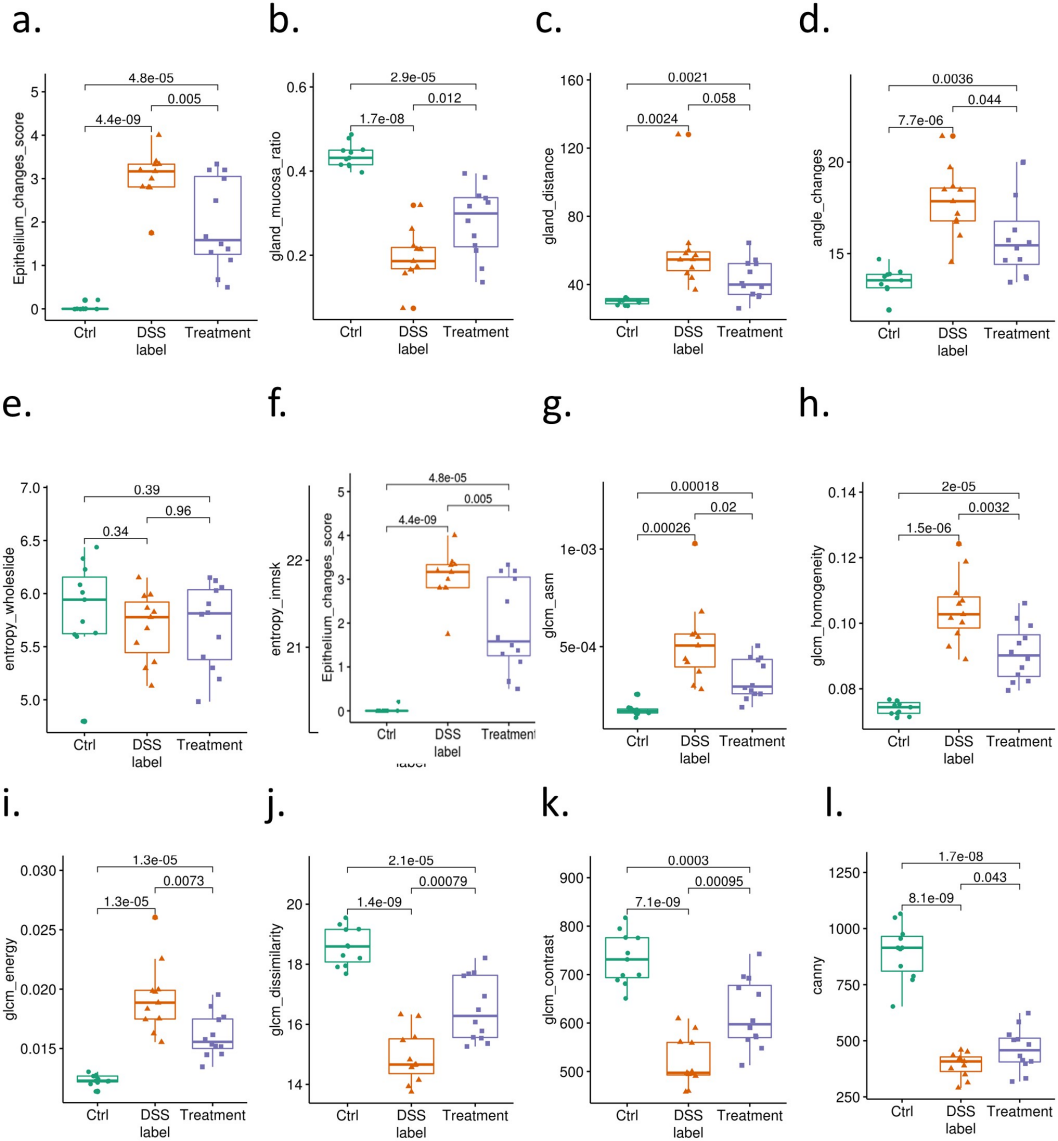
Group comparison among Ctrl (Naïve animal), DSS (DSS-induced colitis), and Treatment (colitis treated with SSZ) at individual subject level. Data were summarized for individual animals. Each sample represents the average value from all slides derived from individual animal in the study. The group information was shown with three colors. a. epithelial change scored by histopathologist, b. ratio of crypt/mucosa area c. distance between two nearest crypts, d. angle changes between two nearest crypts. e: entropy from patches without applying segmentation masks; entropy from unsegmented slides, f: entropy from masked crypt, g-k: GLCM ASM, Homogeneity, energy, dissimilarity, contrast) l: canny.

Based on the segmented crypts and mucosa image patches, we computed several types of features (Fig 8a). Entropy is a first order statistic texture descriptor. The entropy value of the whole slide did not contain any significant differences between the naïve, DSS, and DSS+ sulfasalazine groups (Fig 8e). In contrast, the entropy value within segmented or masked crypts decreased in the DSS group, and increased in the DSS+SSZ group, for both individual mice levels (Fig 8f). This indicates that image segmentation enables the use of first order texture descriptors to perform analysis. Compared to human generated score or some interpretable measurement, GLCM features (Fig 8g–8l) generally have less variability. Therefore, these features may improve statistical power during hypothesis testing. We proposed that in our case, GLCM texture features may be a useful tool to predict tissue damage or the recovery process. We can use them as a surrogate healing marker to screen the initial results from histology data from preclinical model, followed by verification from pathologists.

**Fig 9:**
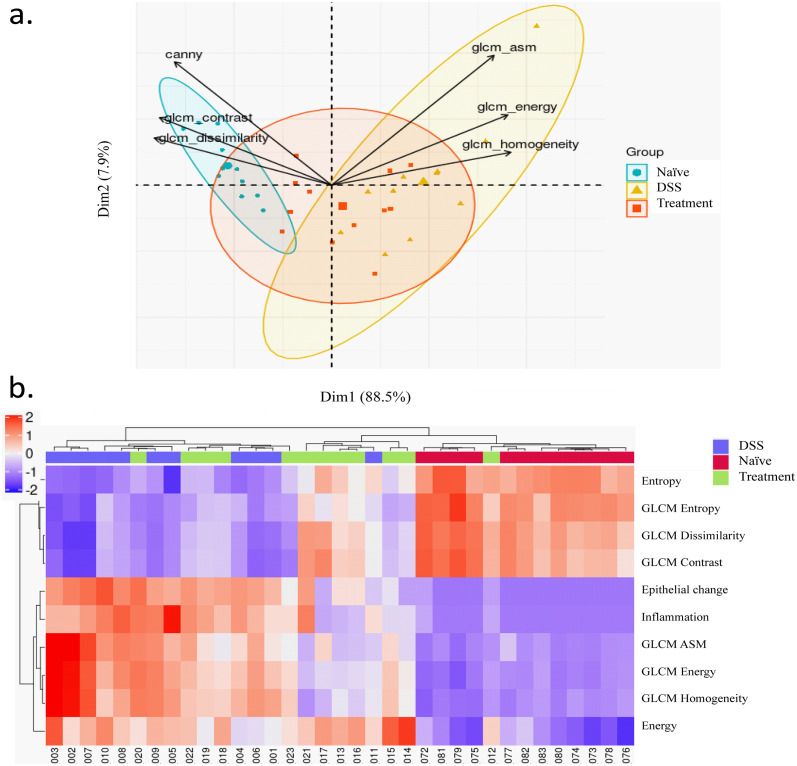
Integration with pathology score. a. PCA plot of crypt image features generated from control, DSS, and SZZ groups were shown. Each dot represents one animal in the study. b. Clusters of samples from Ctrl (Naïve animal), DSS (DSS-induced colitis), and Treatment groups were shown in heatmap using of GLCM features and histopathological scores including epithelium changes and inflammation measurement. Z score normalization was performed.

Our results demonstrate the importance of evaluating both pathologist-interpretable features such as counts, size, and geographical distribution patterns, and subvisual features which are not easy to interpret by humans. The latter category includes first and second order statistical features, reflecting the textural changes of the segmented image. The results of PCA analysis using all the aforementioned features, including histological score for epithelial changes was shown (Fig 9a). The texture features explained the most variation. In addition to statistical features, we included human based epithelial scores and inflammation scores in analysis using the hierarchical clustering method, which relates sample with similar features to each other. In Fig 9b, all the samples from the naïve group forms a cluster, one of the samples from treatment group was also clustered to this group. We confirmed these samples recovered the best among or the treatment group. Compared to the human based score, the combination of texture feature scores also yielded similar results. 7 of 12 samples, which form a cluster in the middle, had already partially recovered in this time point. The results indicate that both human interpretable features and texture features indicate most of the sample had partial recovery in the treatment group. We also propose that analysis of combined features may be helpful for pathologists to adopt more statistical features as a surrogate in practice. In summary, the results of our morphometric feature analysis are consistent with those from the autoencoder visualization: that the histology of mice DSS crypt groups treated with SSZ, differing from DSS-treated tissues, undergo recovery toward normality. The GLCM texture features may be appropriate candidate features to represent current pathology scores. Differentiation of the complex structure like crypts may rely on second order statistical features.

## Discussion

Currently, most of the deep learning applications in biomedical image data analysis serve as a diagnostic aid. Because the criteria of pathology diagnosis is established, the common approach is to create a large amount of labeled training data, which are provided by pathologists, to train a convolutional neural network. However, preclinical therapeutic efficacy testing or in vivo biology research that involves multiple conditions including therapeutics or genetic manipulations, the types of histopathological changes is unknown or unpredictable. Therefore, it is difficult to use the classification model in practice. Here we proposed multiple staged workflows rather then a single deep learning architecture which focus on classification tasks. Image segmentation is a common task that need training data. To fully make use of the limited annotation data, we implemented random tiling generation and image augmentation to make training more efficient. During the training, using bootstrap interactive approaches to correct erroneously segmented structures greatly improve the training efficiency. For tasks that appear to be also difficult to pathologist, we proposed to use autoencoder to help classify and visualize the segmented object for further investigation performed by histopathologists. In addition, a large amount of morphometry information embedded in images is often under-appreciated due to the limited availability of highly skilled pathologists to resolve details in this capacity. From the perspective of computational biology, most of this information is also underutilized. We propose that deep learning based exploratory research will improve the scalability, quantification, and interpretation value for preclinical studies. With this approach, the analysis time can be greatly reduced from weeks to hours. Furthermore, the output data points will increase a thousand times. To implement this application in a pathology lab, our workflow attempted to solve several challenges described in the following paragraphs.

Firstly, a high-performance pipeline is needed to accelerate the training and prediction process. For deep learning using WSIs, the improvement of accuracy may cost a tremendous amount of additional math processing operations with significantly higher demand for computer processing power especially for complicated tasks such as segmentation. We adopted a convolutional U-Net design, which has been the most popular backbone in the medical imaging community due to its impressive result even with a scarce amount of training data.

Secondly, the challenge of the WSIs’ images lie in their large sizes of pixels, densely distributed target object, which vary greatly in their sizes and shapes. Unlike nuclei or cells, crypts are structures that are extremely variable especially in different pathophysiological conditions. Using a colitis model as an example, scoring has been widely used as a method to incorporate multiple coarse grainy measurements to include gland/crypt architecture, degree of inflammatory cell infiltration, muscle thickening, goblet cell depletion and crypt abscess. In this report, we focused on one important structure of crypt glands found in the epithelial lining of the small intestine and colon between the villi. Intestinal crypts contains two types of epithelium cells: mucus-secreting goblet cells and enterocytes that secrete electrolytes and water. For a preclinical study with an exploratory perspective, it is important to survey all possible morphological variants and the full spectrum of histological changes of the targeted tissue or structure. We implemented an autoencoder in order to obtain a representative via unsupervised learning using segmented crypts image obtained from WSIs. The t-SNE visualization of bottleneck from a single autoencoder did not achieve discernable clustering. We used separate channels to randomly sample peripheral region of the segmented crypts as target object, and another one to randomly sample the central region of the crypts. By separate two sets of input, the t-SNE visualization started to show better patterns. This technique enables us to quickly visualize the full spectrum of crypts morphometrics as well as extract typical characteristics. While human’s pathology learning process rely on slides with typical characteristics, as well as their variation in a supervised manner, the autoencoder learns in an unsupervised fashion. Visualize the representative learning result will help us identify novel patterns, which could appear in new models and novel treatment condition. It also facilitates the cross-validation of pathologist’s observation. It makes deep learning results interpretable and help to make differentiation of various therapeutics possible.

Thirdly, interpretation of deep learning result remains challenges to the pathology community.

Crypts mucosa area ratio and the distance between glands is an easy to understand feature since during the DSS treatment, crypts loss occurs and the distance between glands were widened. However, statistical features such as entropy in masked glands, are statistically significant between normal, DSS, and treatment group. Gland feature based analysis strengthen statistical power of the study. One caveat is that value such as entropy could be interpretable differently during a variety of conditions. By incorporating the autoencoder results, we realized a significant portion of glands from DSS are atrophic, or with less cells and lost texture of mucus, which will decrease entropy value significantly. Furthermore, compared to DSS group, glands from treatment group contain more mucus. This explains the recovering of entropy level. Both AE based unsupervised learning and morphometric analysis suggest SSZ group partially restore the crypts architecture in DSS model. The intuitive graphic visualization helps the interpretation of the segmented data, suggesting the necessity to include representative learning information in analysis. We also find the GLCM texture features to be an appropriate test. Differentiation of the complex structure like crypts mostly likely need to rely on second order statistical features.

In summary, the aim of this work was to demonstrate a deep learning powered image analysis workflow to enable preclinical efficacy analysis using intestinal crypts as a proof of concept example. Our workflow is designed for general purpose object segmentation using WSI’s. Different models can be built into the workflow with ease. Implement similar analytic pipeline enable to generate large scale of relevant data from H&E stained WSI’s, which were ubiquitously available in labs related to the histopathology work to accelerate the in vivo preclinical exploratory study. For the limitation of the study, our aim is not to compare different segmentation models. Instead, our workflow is designed for general purpose object segmentation and textual analysis using WSI’s. Different models can be built into the workflow with ease We hope our report will inspire more efforts in the research field using modern, scalable quantitative automated processes and deep learning algorithms to reduce time, costs, and improve resource allocation efficiency in drug discovery. We hope our report will inspire more efforts in the research field using modern, scalable quantitative automated processes and deep learning algorithms to reduce time, costs, and improve resource allocation efficiency in drug discovery.

## Conclusion

Digitalization of histopathology in WSIs provides a new source of data that will help pathologists better understand the therapeutic effects at cellular levels. We proposed a computational workflow to make large quantity of image features, once being too labour intensive to manually capture, become available and ready to explore for in vivo model based preclinical study. Through a deep learning approach, quantitative analysis of histological remission can be automated, reducing the time required from a matter of weeks to hours, increasing the volume of data that can be analyzed with minimal human supervision.

## Supporting information

S1 Appendix (PDF)

## References

[1] Tomoyose M, Mitsuyama K, Ishida H, Toyonaga A, Tanikawa K. Role of Interleukin-10 in a Murine Model of Dextran Sulfate Sodium-Induced Colitis. Scand J Gastroentero. 2009;33: 435–440. doi: 10.1080/00365529850171080 9605267

[2] Kersting S, Behrendt V, Kersting J, Reinecke K, Hilgert C, Stricker I, et al. The impact of JNK inhibitor D-JNKI-1 in a murine model of chronic colitis induced by dextran sulfate sodium. J Inflamm Res. 2013;6: 71–81. doi: 10.2147/JIR.S40092 23667316 PMC3650567

[3] Charlet R, Sendid B, Kaveri SV, Poulain D, Bayry J, Jawhara S. Intravenous Immunoglobulin Therapy Eliminates Candida albicans and Maintains Intestinal Homeostasis in a Murine Model of Dextran Sulfate Sodium-Induced Colitis. Int J Mol Sci. 2019;20: 1473. doi: 10.3390/ijms20061473 30909599 PMC6471409

[4] Kabashima K, Saji T, Murata T, Nagamachi M, Matsuoka T, Segi E, et al. The prostaglandin receptor EP4 suppresses colitis, mucosal damage and CD4 cell activation in the gut. J Clin Invest. 2002;109: 883–893. doi: 10.1172/JCI14459 11927615 PMC150928

[5] Sann H, von Erichsen J, Hessmann M, Pahl A, Hoffmeyer A. Efficacy of drugs used in the treatment of IBD and combinations thereof in acute DSS-induced colitis in mice. Life Sci. 2013;92: 708–718. doi: 10.1016/j.lfs.2013.01.028 23399699

[6] Kim JJ, Shajib MS, Manocha MM, Khan WI. Investigating Intestinal Inflammation in DSS-induced Model of IBD. J Vis Exp. 2012. doi: 10.3791/3678 22331082 PMC3369627

[7] Eichele DD, Kharbanda KK. Dextran sodium sulfate colitis murine model: An indispensable tool for advancing our understanding of inflammatory bowel diseases pathogenesis. World J Gastroentero. 2017;23: 6016–6029. doi: 10.3748/wjg.v23.i33.6016 28970718 PMC5597494

[8] Erben U, Loddenkemper C, Doerfel K, Spieckermann S, Haller D, Heimesaat MM, et al. A guide to histomorphological evaluation of intestinal inflammation in mouse models. Int J Clin Exp Patho. 2014;7: 4557–76. 25197329 PMC4152019

[9] Wang Y, Chiang I-L, Ohara TE, Fujii S, Cheng J, Muegge BD, et al. Long-Term Culture Captures Injury-Repair Cycles of Colonic Stem Cells. Cell. 2019;179: 1144–1159.e15. doi: 10.1016/j.cell.2019.10.015 31708126 PMC6904908

[10] Rathore S, Iftikhar MA, Chaddad A, Niazi T, Karasic T, Bilello M. Segmentation and Grade Prediction of Colon Cancer Digital Pathology Images Across Multiple Institutions. Cancers. 2019;11: 1700. doi: 10.3390/cancers11111700 31683818 PMC6896042

[11] Xu J, Monaco JP, Sparks R, Madabhushi A. Connecting Markov random fields and active contour models: application to gland segmentation and classification. J Medical Imaging. 2017;4: 021107–021107. doi: 10.1117/1.JMI.4.2.021107 28382316 PMC5369422

[12] Xu J, Janowczyk A, Chandran S, Madabhushi A. A high-throughput active contour scheme for segmentation of histopathological imagery. Med Image Anal. 2011;15: 851–862. doi: 10.1016/j.media.2011.04.002 21570336 PMC3168681

[13] Graham S, Chen H, Gamper J, Dou Q, Heng P-A, Snead D, et al. MILD-Net: Minimal information loss dilated network for gland instance segmentation in colon histology images. Med Image Anal. 2019;52: 199–211. doi: 10.1016/j.media.2018.12.001 30594772

[14] Xu Y, Li Y, Wang Y, Liu M, Fan Y, Lai M, et al. Gland Instance Segmentation Using Deep Multichannel Neural Networks. Ieee T Bio-med Eng. 2017;64: 2901–2912. doi: 10.1109/TBME.2017.2686418 28358671

[15] Binder T, Tantaoui EM, Pati P, Catena R, Set-Aghayan A, Gabrani M. Multi-Organ Gland Segmentation Using Deep Learning. Frontiers Medicine. 2019;6: 173. doi: 10.3389/fmed.2019.00173 31428614 PMC6690405

[16] Sirinukunwattana K, Pluim JPW, Chen H, Qi X, Heng P-A, Guo YB, et al. Gland segmentation in colon histology images: The glas challenge contest. Med Image Anal. 2017;35: 489–502. doi: 10.1016/j.media.2016.08.008 27614792

[17] Chen H, Qi X, Yu L, Dou Q, Qin J, Heng P-A. DCAN: Deep contour-aware networks for object instance segmentation from histology images. Med Image Anal. 2017;36: 135–146. doi: 10.1016/j.media.2016.11.004 27898306

[18] Lin T-Y, Dollár P, Girshick R, He K, Hariharan B, Belongie S. Feature Pyramid Networks for Object Detection. 2017 Ieee Conf Comput Vis Pattern Recognit Cvpr. 2017; 936–944. doi: 10.1109/cvpr.2017.106

[19] Korfhage N, Mühling M, Ringshandl S, Becker A, Schmeck B, Freisleben B. Detection and segmentation of morphologically complex eukaryotic cells in fluorescence microscopy images via feature pyramid fusion. Plos Comput Biol. 2020;16: e1008179. doi: 10.1371/journal.pcbi.1008179 32898132 PMC7523959

[20] Zhao H, Shi J, Qi X, Wang X, Jia J. Pyramid Scene Parsing Network. 2017 Ieee Conf Comput Vis Pattern Recognit Cvpr. 2017; 6230–6239. doi: 10.1109/cvpr.2017.660

[21] Hira S, Bai A, Hira S. An automatic approach based on CNN architecture to detect Covid-19 disease from chest X-ray images. Appl Intell. 2020; 1–26. doi: 10.1007/s10489-020-02010-w PMC7693857 34764572

[22] Miao R, Toth R, Zhou Y, Madabhushi A, Janowczyk A. Quick Annotator: an open-source digital pathology based rapid image annotation tool. Arxiv. 2021. 10.1002/cjp2.229 PMC8503896 34288586

