# Supplementary material for: Deep learning for discovering pathological continuum of crypts and evaluating therapeutic effects: An implication for in vivo preclinical study
Source: PLoS One. 2021 Jun 14;16(6):e0252429. doi: 10.1371/journal.pone.0252429 (PMC8202954; doi:10.1371/journal.pone.0252429)
Supplement: S1 Appendix — (PDF) [file pone.0252429.s001.pdf]

PLoS One S1 Appendix

Article title: Deep Learning for Discovering Pathological Continuum of Crypts and Evaluating Therapeutic Effects: an Implication for in vivo Preclinical Study

Authors: Dechao Shan, Jie Zheng, Alexander Klimowicz, Mark Panzenbeck, Zheng Liu, Di Feng

The following Supporting Information is available for this article:

S1 Fig.A. Random resized crop for spatial augmentation
Fig.B. The effect of spatial segmentation on F1 score
Fig.C. Comparison with specialized model from Glas contest
Fig.D. Segmentation model parameters
Fig.E. Model accuracy curve comparison
Fig.F. Model comparison using F1 score
Fig.G. Accuracy curve comparison using partially trained model
Fig.H. F1 score using partially trained model
Fig.I. U-Net model summary
Fig.J. Autoencoder summary

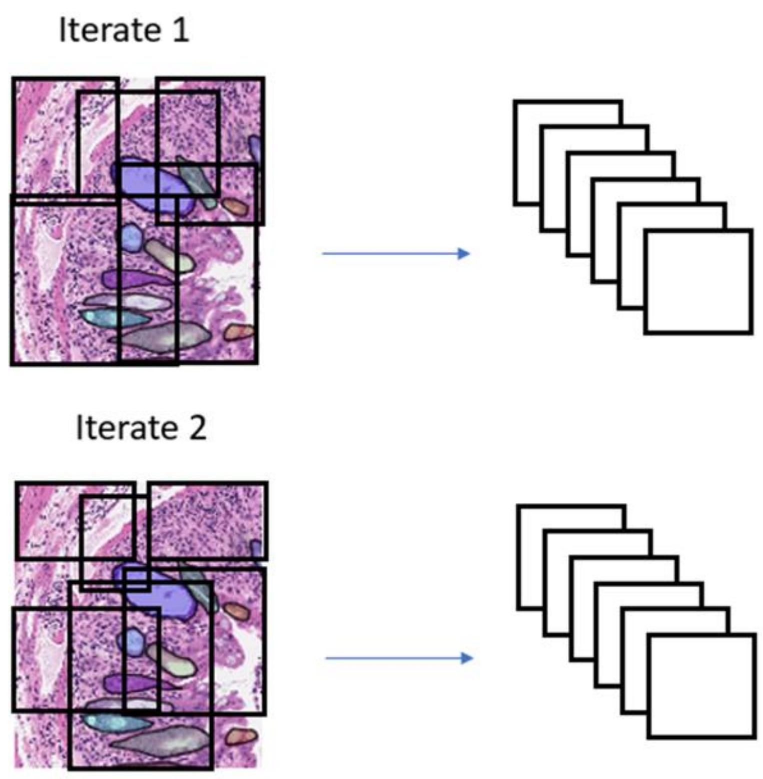

A) The process of random resized crop for spatial augmentation. For each iteration, crop of random size of the original size in range 256-768 with a random aspect ratio of the original, followed by final resize to 512X512. Each iterate can generate different tiles as training set.

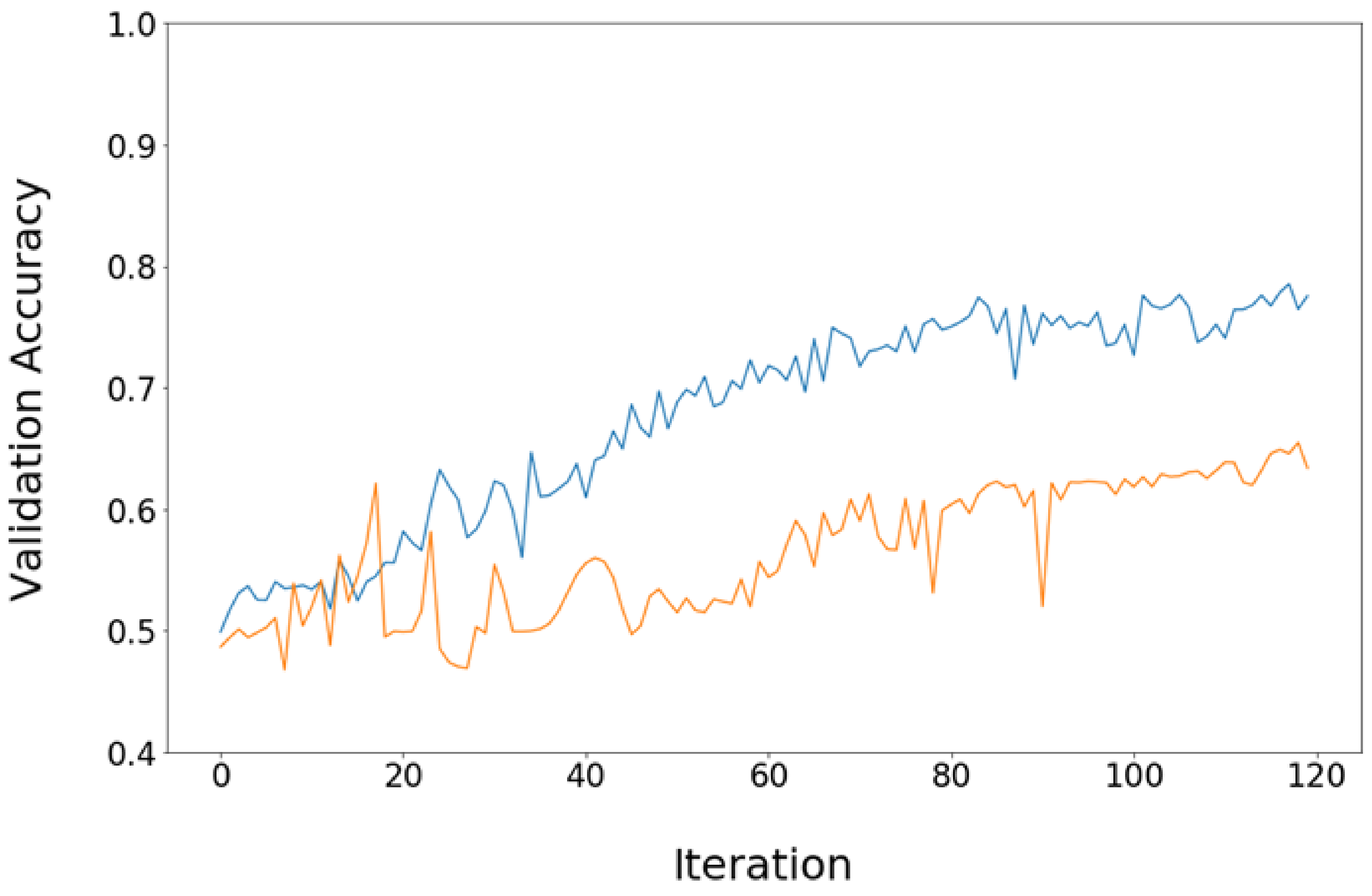

B) To show the effect of spatial augmentation using random resized crop, we used 30% of the GlaS dataset: training data to train the U-Net and generate accuracy curve using all validation data. The orange line represent accuracy curve from model with no spatial augmentation. The blue line indicate the accuracy curve with augmentation.

Model	F1	Dice
U-Net	0.872	0.86
MILD-Net	0.82	0.836
CUMedVision1	0.769	0.8
ExB3	0.719	0.765
CUMedVision2	0.716	0.781
ExB1	0.703	0.786

C) Comparative analysis of top ranked models on the GlaS challenge dataset. F1 and DICE Scores from top 4 models reported from Glas contest and MILD models were used to compare with our implementation of U-Net using part B validation datasets.

Model	Backbone	Input shape	Output classes	Encoder weight	Activation	Others
PSP	resnet18	Divisible by down sample factor	1	None	Sigmoid	Pooling: average
LinkNet	resnet18	Pass original	1	None	Sigmoid	Decode: upsampling
FPN	resnet18	Pass original	1	None	Sigmoid	Pyramid aggregation: ‘concat’
Unet	resnet18	Pass original	1	None	Sigmoid	Decode: upsampling

D). The Python segmentation-models API parameters used for comparison of general segmentation models used in the following C-F. The listed parameters reflected the model with comparable implementation. For other parameters, default values were used.

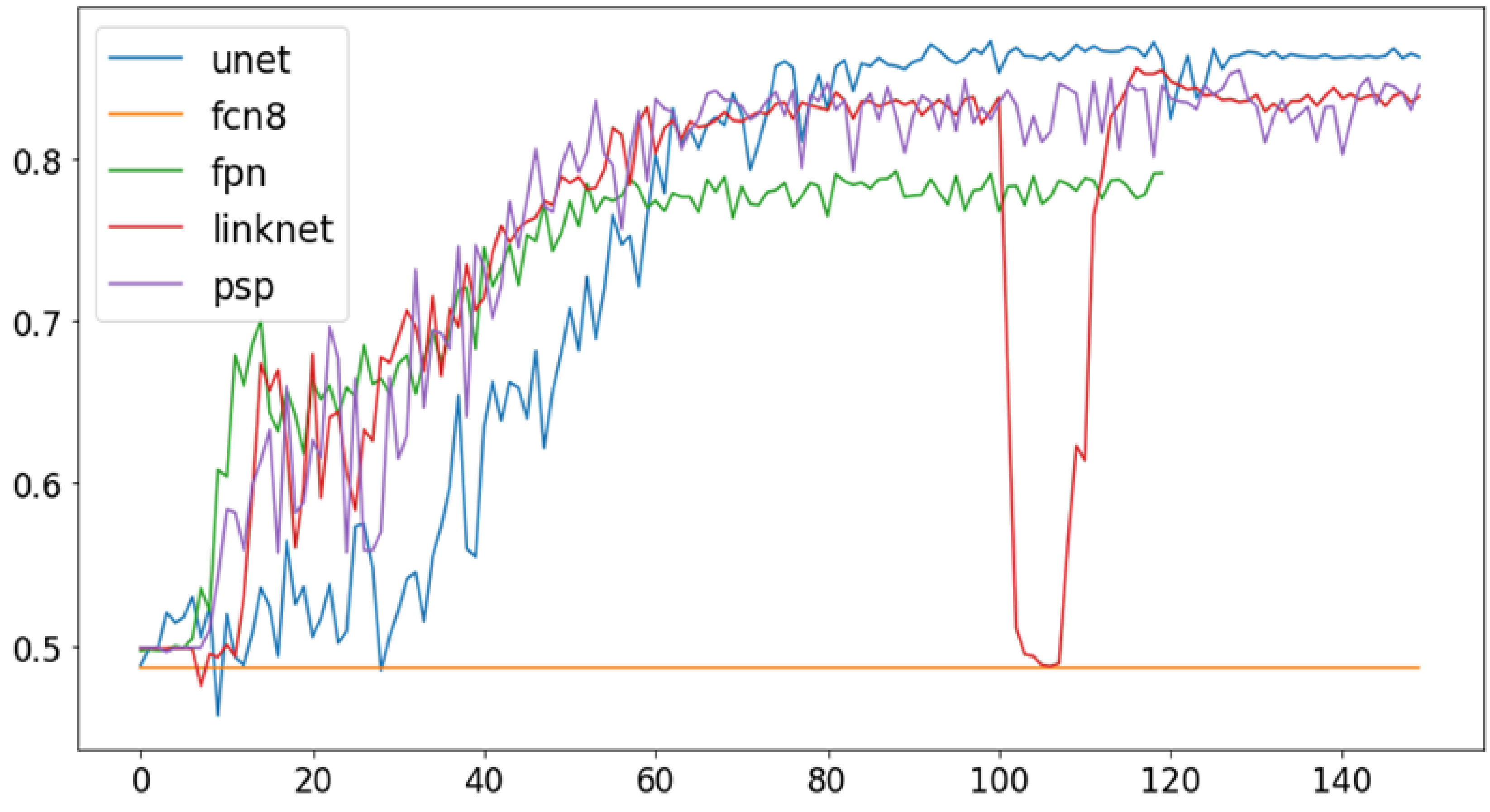

E) We compared the accuracy our UNet implementation with other four different models including fcn8,fpn, linknet, and psp. Accuracy curve using GlaS contest validation dataset were shown in different color. Out implementation of UNnet reach good performance after 80 epochs.

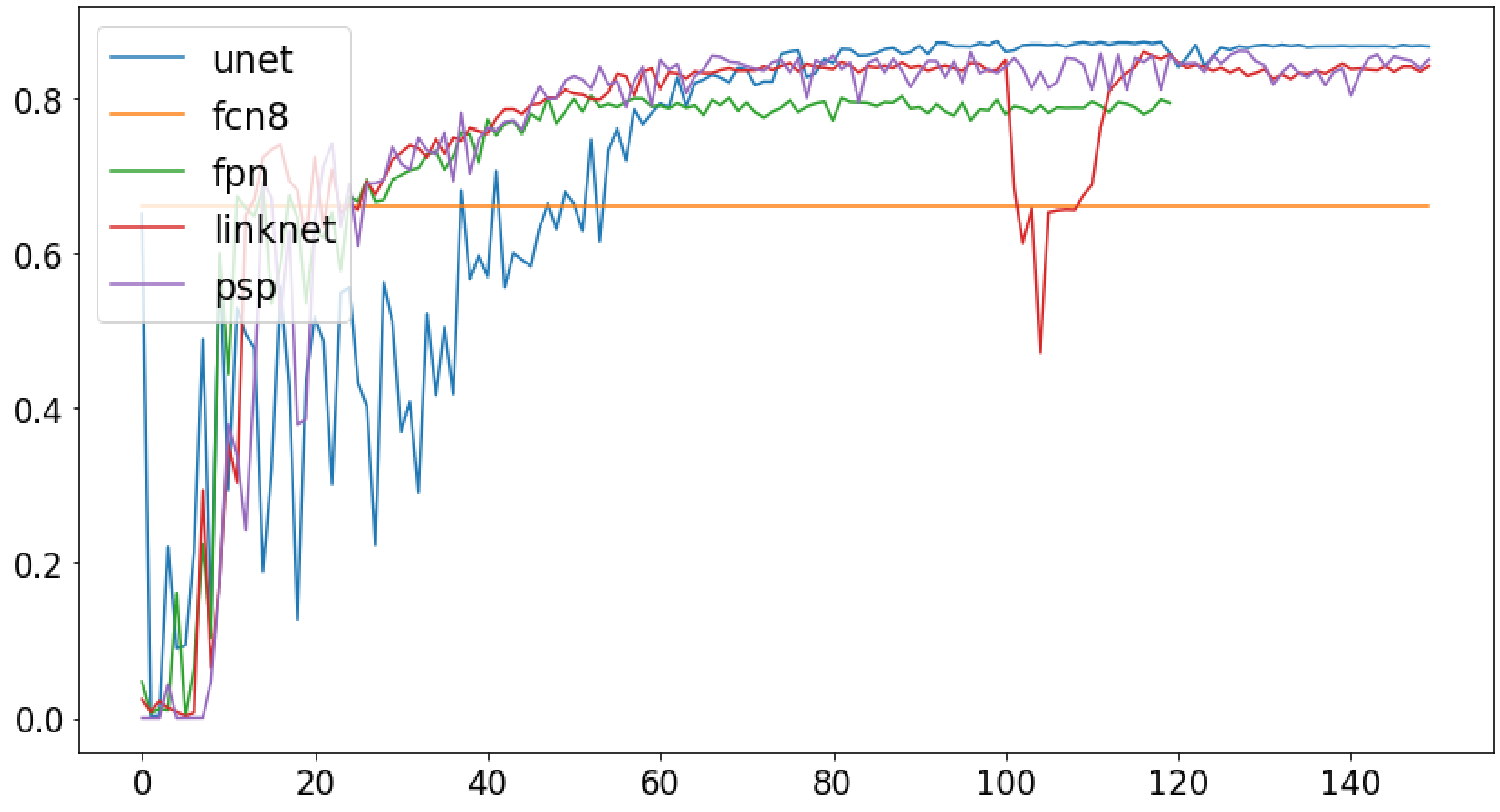

F) Comparison of F1 score using U-Net, FCN8, FPN Linknet, and PSP. Accuracy curve using GlaS contest validation dataset were shown in different color. Our implementation of U-Net reached good performance after 80 epochs.

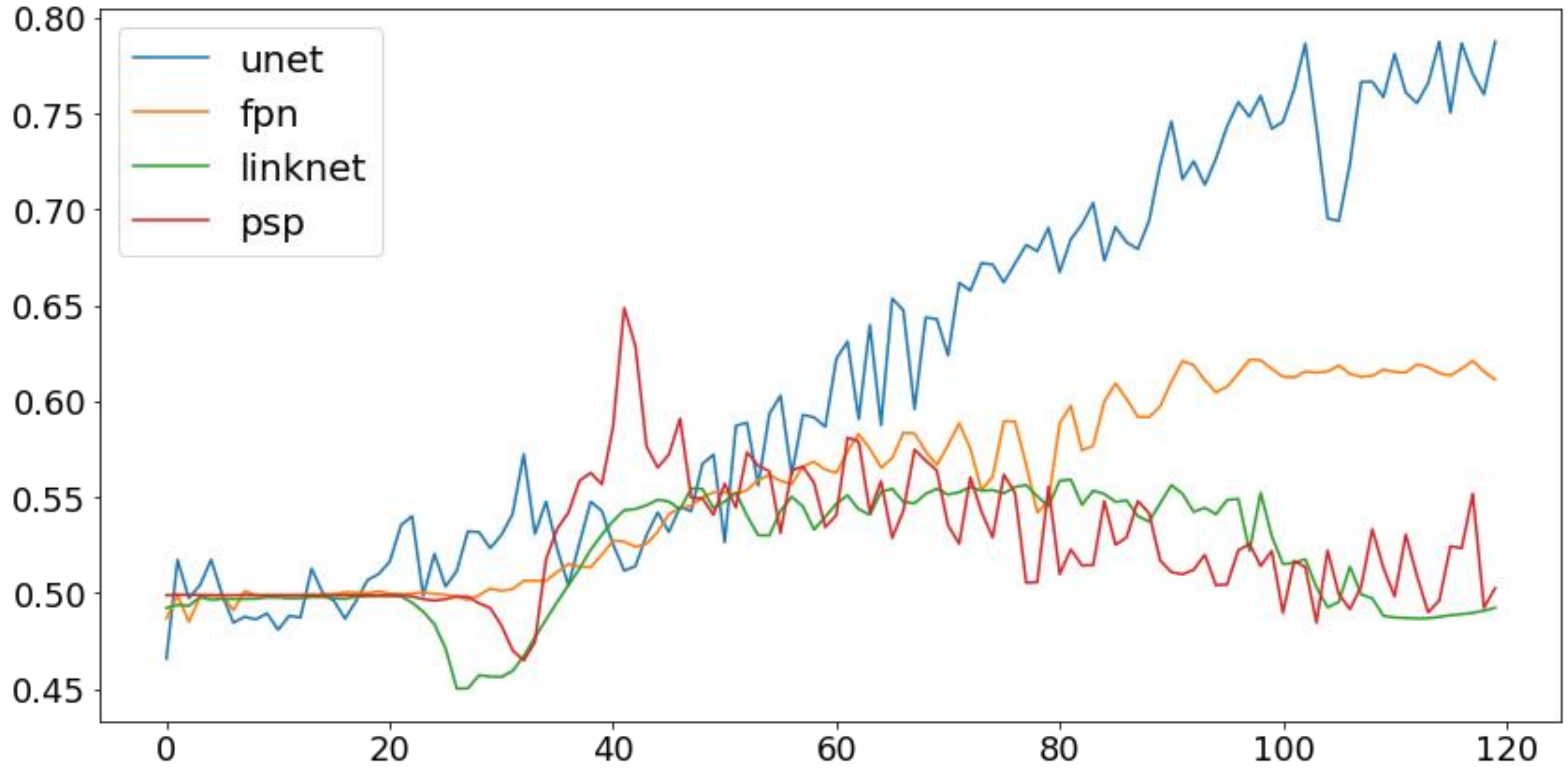

G) We compared accuracy from UNet implementation with other four different models including fcn8,fpn,linknet, and psp. All models were trained using 30% of the training set drawn from glas dataset. F1 score curve using all validation dataset were shown in different color. Out implementation of UNet reach superior performance after 60 epochs when smaller number of training set was used.

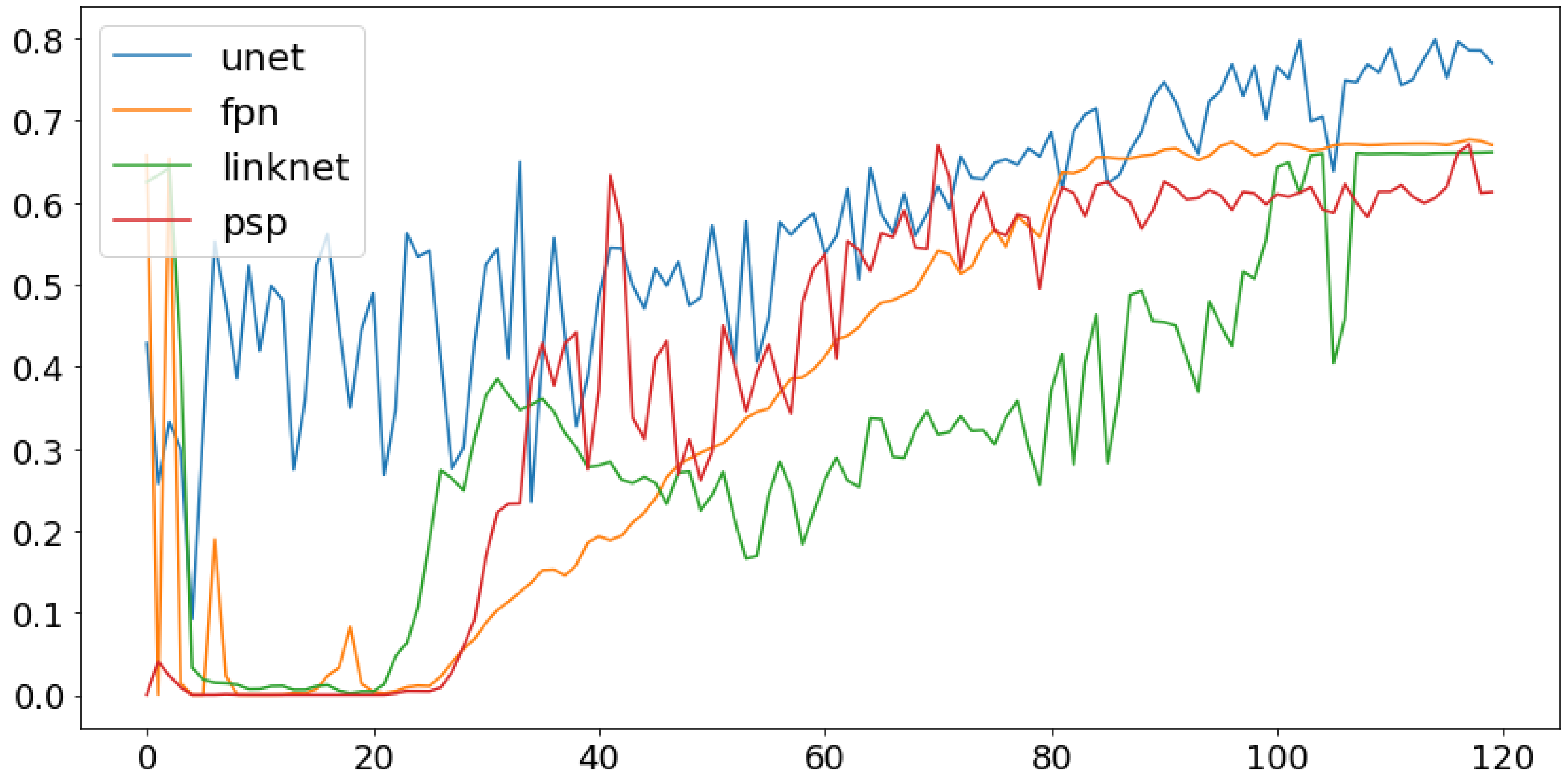

H) We compared F1 score from UNet implementation with other four different models including fcn8,fpn,linknet, and psp. All models were trained using 30% of the training set. F1 score curve using all validation dataset were shown in different color. Out implementation of UNet reach good performance after 60 epochs when smaller number of training set was used.

I) U-Net Implementation Summary

```
Model: UNet
Layer (type)                  Output Shape         Param #   Connected to
==================================================================================================
input_1 (InputLayer)          [(None, 512, 512, 3) 0
__________________________________________________________________________________________________
conv2d (Conv2D)               (None, 512, 512, 16) 448       input_1[0][0]
__________________________________________________________________________________________________
conv2d_1 (Conv2D)             (None, 512, 512, 16) 2320      conv2d[0][0]
__________________________________________________________________________________________________
max_pooling2d (MaxPooling2D)  (None, 256, 256, 16) 0         conv2d_1[0][0]
__________________________________________________________________________________________________
conv2d_2 (Conv2D)             (None, 256, 256, 32) 4640      max_pooling2d[0][0]
__________________________________________________________________________________________________
conv2d_3 (Conv2D)             (None, 256, 256, 32) 9248      conv2d_2[0][0]
__________________________________________________________________________________________________
max_pooling2d_1 (MaxPooling2D)  (None, 128, 128, 32) 0       conv2d_3[0][0]
__________________________________________________________________________________________________
conv2d_4 (Conv2D)             (None, 128, 128, 64) 18496     max_pooling2d_1[0][0]
__________________________________________________________________________________________________
conv2d_5 (Conv2D)             (None, 128, 128, 64) 36928     conv2d_4[0][0]
__________________________________________________________________________________________________
max_pooling2d_2 (MaxPooling2D)  (None, 64, 64, 64)   0       conv2d_5[0][0]
__________________________________________________________________________________________________
conv2d_6 (Conv2D)             (None, 64, 64, 128)  73856     max_pooling2d_2[0][0]
__________________________________________________________________________________________________
conv2d_7 (Conv2D)             (None, 64, 64, 128)  147584    conv2d_6[0][0]
__________________________________________________________________________________________________
max_pooling2d_3 (MaxPooling2D)  (None, 32, 32, 128)  0       conv2d_7[0][0]
__________________________________________________________________________________________________
conv2d_8 (Conv2D)             (None, 32, 32, 256)  295168    max_pooling2d_3[0][0]
__________________________________________________________________________________________________
conv2d_9 (Conv2D)             (None, 32, 32, 256)  590080    conv2d_8[0][0]
__________________________________________________________________________________________________
max_pooling2d_4 (MaxPooling2D)  (None, 16, 16, 256)  0       conv2d_9[0][0]
__________________________________________________________________________________________________
conv2d_10 (Conv2D)            (None, 16, 16, 512)  1180160   max_pooling2d_4[0][0]
__________________________________________________________________________________________________
conv2d_11 (Conv2D)            (None, 16, 16, 512)  2359808   conv2d_10[0][0]
__________________________________________________________________________________________________
up_sampling2d (UpSampling2D)  (None, 32, 32, 512)  0         conv2d_11[0][0]
__________________________________________________________________________________________________
concatenate (Concatenate)     (None, 32, 32, 768)  0         up_sampling2d[0][0]
                                                             conv2d_9[0][0]
__________________________________________________________________________________________________
conv2d_12 (Conv2D)            (None, 32, 32, 256)  1769728   concatenate[0][0]
__________________________________________________________________________________________________
conv2d_13 (Conv2D)            (None, 32, 32, 256)  590080    conv2d_12[0][0]
__________________________________________________________________________________________________
up_sampling2d_1 (UpSampling2D)  (None, 64, 64, 256)  0       conv2d_13[0][0]
__________________________________________________________________________________________________
concatenate_1 (Concatenate)   (None, 64, 64, 384)  0         up_sampling2d_1[0][0]
                                                             conv2d_7[0][0]
__________________________________________________________________________________________________
conv2d_14 (Conv2D)            (None, 64, 64, 128)  442496    concatenate_1[0][0]
__________________________________________________________________________________________________
conv2d_15 (Conv2D)            (None, 64, 64, 128)  147584    conv2d_14[0][0]
__________________________________________________________________________________________________
up_sampling2d_2 (UpSampling2D)  (None, 128, 128, 128 0       conv2d_15[0][0]
__________________________________________________________________________________________________
concatenate_2 (Concatenate)   (None, 128, 128, 192 0         up_sampling2d_2[0][0]
                                                             conv2d_5[0][0]
__________________________________________________________________________________________________
conv2d_16 (Conv2D)            (None, 128, 128, 64) 110656    concatenate_2[0][0]
__________________________________________________________________________________________________
conv2d_17 (Conv2D)            (None, 128, 128, 64) 36928     conv2d_16[0][0]
__________________________________________________________________________________________________
up_sampling2d_3 (UpSampling2D)  (None, 256, 256, 64) 0       conv2d_17[0][0]
__________________________________________________________________________________________________
concatenate_3 (Concatenate)   (None, 256, 256, 96) 0         up_sampling2d_3[0][0]
                                                             conv2d_3[0][0]
__________________________________________________________________________________________________
conv2d_18 (Conv2D)            (None, 256, 256, 32) 27680     concatenate_3[0][0]
__________________________________________________________________________________________________
conv2d_19 (Conv2D)            (None, 256, 256, 32) 9248      conv2d_18[0][0]
__________________________________________________________________________________________________
up_sampling2d_4 (UpSampling2D)  (None, 512, 512, 32) 0       conv2d_19[0][0]
__________________________________________________________________________________________________
concatenate_4 (Concatenate)   (None, 512, 512, 48) 0         up_sampling2d_4[0][0]
                                                             conv2d_1[0][0]
__________________________________________________________________________________________________
conv2d_20 (Conv2D)            (None, 512, 512, 16) 6928      concatenate_4[0][0]
__________________________________________________________________________________________________
conv2d_21 (Conv2D)            (None, 512, 512, 16) 2320      conv2d_20[0][0]
__________________________________________________________________________________________________
conv2d_22 (Conv2D)            (None, 512, 512, 1)  17        conv2d_21[0][0]
==================================================================================================
Total params: 7,862,401
Trainable params: 7,862,401
Non-trainable params: 0
```

J) Autoencoder Implementation Summary

```
Model: Autoencoder
Layer (type)                 Output Shape              Param #
=================================================================i
nput_1 (InputLayer)          [(None, 24, 24, 72)]      0
conv2d (Conv2D)              (None, 24, 24, 96)        7008
conv2d_1 (Conv2D)            (None, 24, 24, 96)        83040
max_pooling2d (MaxPooling2D) (None, 12, 12, 96)        0
conv2d_2 (Conv2D)            (None, 12, 12, 64)        6208
conv2d_3 (Conv2D)            (None, 12, 12, 64)        36928
max_pooling2d_1 (MaxPooling2 (None, 6, 6, 64)          0
conv2d_4 (Conv2D)            (None, 6, 6, 32)          2080
conv2d_5 (Conv2D)            (None, 6, 6, 32)          9248
max_pooling2d_2 (MaxPooling2 (None, 3, 3, 32)          0
conv2d_6 (Conv2D)            (None, 3, 3, 24)          6936
conv2d_7 (Conv2D)            (None, 3, 3, 24)          5208
up_sampling2d (UpSampling2D) (None, 6, 6, 24)          0
conv2d_8 (Conv2D)            (None, 6, 6, 32)          800
conv2d_9 (Conv2D)            (None, 6, 6, 32)          9248
up_sampling2d_1 (UpSampling2 (None, 12, 12, 32)        0
conv2d_10 (Conv2D)           (None, 12, 12, 64)        2112
conv2d_11 (Conv2D)           (None, 12, 12, 64)        36928
up_sampling2d_2 (UpSampling2 (None, 24, 24, 64)        0
conv2d_12 (Conv2D)           (None, 24, 24, 96)        6240
conv2d_13 (Conv2D)           (None, 24, 24, 96)        83040
conv2d_14 (Conv2D)           (None, 24, 24, 72)        6984
=================================================================
Total params: 302,008Trainable params: 302,008Non-trainable params:0
```
